# *Listeria monocytogenes* Biofilm Adaptation to Different Temperatures Seen Through Shotgun Proteomics

**DOI:** 10.3389/fnut.2019.00089

**Published:** 2019-06-14

**Authors:** Tiago Santos, Didier Viala, Christophe Chambon, Julia Esbelin, Michel Hébraud

**Affiliations:** ^1^Université Clermont Auvergne, INRA, UMR Microbiologie Environnement Digestif Santé (MEDiS), Saint-Genès-Champanelle, France; ^2^INRA, Plateforme d'Exploration du Métabolisme, Composante Protéomique (PFEMcp), Saint-Genès-Champanelle, France

**Keywords:** *Listeria monocytogenes*, temperature adaptation, biofilm, subproteomes, shotgun proteomics

## Abstract

*Listeria monocytogenes* is a foodborne pathogen that can cause invasive severe human illness (listeriosis) in susceptible patients. Most human listeriosis cases appear to be caused by consumption of refrigerated ready-to-eat foods. Although initial contamination levels in foods are usually low, the ability of these bacteria to survive and multiply at low temperatures allows it to reach levels high enough to cause disease. This study explores the set of proteins that might have an association with *L. monocytogenes* adaptation to different temperatures. Cultures were grown in biofilm, the most widespread mode of growth in natural and industrial realms. Protein extractions were performed from three different growth temperatures (10, 25, and 37°C) and two growth phases (early stage and mature biofilm). *L. monocytogenes* subproteomes were targeted using three extraction methods: trypsin-enzymatic shaving, biotin-labeling and cell fractionation. The different subproteomes obtained were separated and analyzed by shotgun proteomics using high-performance liquid chromatography combined with tandem mass spectrometry (LC-OrbiTrap LTQVelos, ThermoFisher Scientific). A total of 141 (biotinylation), 98 (shaving) and 910 (fractionation) proteins were identified. Throughout the 920 unique proteins identified, many are connected to basic cell functions, but some are linked with thermoregulation. We observed some noteworthy protein abundance shifts associated with the major adaptation to cold mechanisms present in *L. monocytogenes*, namely: the role of ribosomes and the stressosome with a higher abundance of the general stress protein Ctc (Rl25) and the general stress transcription factor sigma B (σ^B^), changes in cell fluidity and motility seen by higher levels of foldase protein PrsA2 and flagellin (FlaA), the uptake of osmolytes with a higher abundance of glycine betaine (GbuB) and carnitine transporters (OpucA), and the relevance of the overexpression of chaperone proteins such as cold shock proteins (CspLA and Dps). As for 37°C, we observed a significantly higher percentage of proteins associated with transcriptional or translational activity present in higher abundance upon comparison with the colder settings. These contrasts of protein expression throughout several conditions will enrich databases and help to model the regulatory circuitry that drives adaptation of *L. monocytogenes* to environments.

## Introduction

The ready-to-eat (RTE) food sector is in constant expansion, offering a wide variety and number of products to consumers. Unfortunately, with that comes an increased chance of microbial contamination, as is the case of *Listeria monocytogenes* (1), a Gram-positive foodborne pathogen bacterium and the causative agent of the illness listeriosis (2). The European Food Safety Authority (EFSA) reported, in Europe, from 2008 to 2015, 37 food-borne outbreaks caused by *L. monocytogenes* that lead to 37 deaths (3). Just from June of 2018, 47 cases have been reported, and nine patients have died due to or with the infection (4). Even if the annual number of listeriosis cases comes behind other major foodborne pathogens (~23,150 listeriosis cases were estimated worldwide in 2010), the mortality among infected individuals is very high, reaching levels up to 30% (5). This ubiquitous biofilm-forming bacterium is found throughout the environment, including soil, vegetation, and animals (6). The agroecosystems play a major role in the spread of such pathogens in the food chain through the production of contaminated raw products (7).

Bacterial cells are often found in complex communities, termed biofilms, that provide resources and protection to harsh environments (8). The trifactor, that includes pathogenic power, ability to form biofilm and ubiquity qualify *L. monocytogenes* as huge risk for human health. Remarkably, most stress-related reports were performed in planktonic cultures (9), even if there is a clear need to study the bacterial responses associated with stress tolerance and the features and benefits conferred by the sessile mode of growth to bacterial cells. One other *L. monocytogenes* attribute is its proteosurfaceome. Surface proteins are the link between bacteria and its environment, playing a significant role in communication, chemical sensing, stress resistance and balance of nutrients and toxins in the cell (10). *L. monocytogenes* genome sequence revealed 133 genes coding for surface proteins. Notably, the phylogenetically close but non-pathogenic *L. innocua* genome presented despair results regarding this protein family, unveiling for the first time its potential role in virulence (11). Even with the clear importance of proteomes and proteosurfaceome, protein studies have a higher level of complexity in comparison to DNA or RNA, mainly due to its variations in abundance, physicochemical features and subcellular localization (10). As for surface proteins, these are especially hard to work, since they require a carefully balanced hydrophilic and lipophilic environment (12). In regards to the role of surface proteins in virulence, the *L. monocytogenes* proteosurfaceome has been well explored (13), but the same cannot be said for its part in the adaptation and resistance to different environmental settings. Even scarcer are the studies in the sessile mode of growth, the predominant growth state in food workshops (14). To overcome the challenges associated with properly identifying surface-associated proteins, three different but complementary extraction methods were used in this study. First, the biotinylation method which is based on the treatment of intact cells with sulfo-NHS-SS-Biotin, to which the cell membrane is impermeable. This marker molecule reacts specifically with the ε-amino-group of lysine residues of surface-exposed proteins. Subsequently, labeled proteins can be separated from non-labeled proteins by affinity chromatography with neutravidin and then analyzed by liquid chromatography-tandem mass spectrometry (LC-MS/MS) (15). Secondly, the shaving method which consists on treating intact bacterial cells with proteases in an isotonic solution to promote the release exposed peptides (16). The third and final extraction method, the fractionation which allows to explore the proteome in separated subcellular fractions.

*L. monocytogenes* is an opportunistic bacterial pathogen that has the capacity to survive under extreme environmental conditions encountered in nature and in the food chain, such as high salt concentrations (17), large range of pH (18), desiccation (19, 20), and low temperatures (21). Maintaining the cold chain is an essential parameter throughout the processing and distribution of food, protecting it from the growth of mesophilic microorganisms and thus extending its shelflife. However, the temperatures used for refrigerated storage do not prevent the growth of psychrotrophic germs such as *L. monocytogenes* (22). Even if cold environments lead to a decrease in the rate of bacterial growth, they do not inhibit it completely (21). Temperature variation also has its role in virulence. As for many other bacterial pathogens, *L. monocytogenes* activates the expression of virulence genes at host body temperature (23). Throughout the years, multiple studies have been published exploring *Listeria*'s temperature adaptation, either through physicochemical tests (21, 24–29), genomic and transcriptomic methods (30–38), and also through metabolic and proteomic approaches (39–44). However, there is still a knowledge gap in the comparison of subproteomic changes to different temperatures, particularly in the biofilm mode of growth.

*L. monocytogenes* adaptation to low temperatures is one of its crucial attributes that supports *Listeria* persistence and dissemination in refrigerated products. This adaptation to cold temperatures renders the use of such physical setting insufficient for the control of *L. monocytogenes* presence in long-term storage under refrigeration products (45). *L. monocytogenes* is able to growth at temperatures as low as −0.4°C but also survive in freezing temperature such as −18°C (21, 46). In short, upon exposure to low temperatures, bacterial membranes become more rigid and the metabolic rate decreases. To overcome the hurdles imposed by a cold stress, bacteria have to increase the expression of genes involved in cell membrane function, production of cold shock proteins and multiple other molecular strategies to maintain homeostasis (47).

One of the major adaptative strategies is the induction of osmolyte and peptide transporters which will increase the amount of this molecules in the cytosol and maintain turgor pressure (48). The critical uptake of osmoprotectant molecules for *Listeria*'s adaptation to low temperature is made through auto transporters for compatible osmolytes and short oligopeptides (49). The main carnitine transporter, OpuC, encoded by the opuCABCD operon, was observed in high abundance in *Listeria* cells exposed to low temperatures (48, 50).

In cold environments, bacteria also duel with protein damage, particularly protein misfolding and aggregation. To counteract this damage, cells have at their disposal a network of molecular chaperones that assist in maintaining proteins in their native states. A key role of chaperones is preventing protein aggregation (51). *L. monocytogenes* harbors three proteins from the CspA family (CspLA, CspLB, and CspD) and genome-wide expression studies showed a significant increase in expression of CspL at low temperatures (52).

*L. monocytogenes* response to the ever-changing environmental factors and stress conditions is linked to the activation of the alternative sigma factor sigma B (σ^B^) that controls the general stress response (GSR). Functions of genes regulated by this transcriptional factor include a diversity of metabolic pathways, transport associated proteins, stress proteins, and other transcriptional factors (53). Particularly the osmolyte carnitine, which as mentioned before is transported via OpuC. The expression of this autotransporter is also regulated by σ^B^. Other genes associated with cold stress resistance, *ltrC* (54) and *fri* (55), are also controlled by σ^B^ regulon. In short, even if a direct link between cryotolerance and σ^B^ has not yet been obtained, there are clear signs that this transcriptional factor has a role in *Listeria*'s adaptation to low temperature (47).

A combination with other mechanisms of cellular homeostasis maintenance, such as: control of membrane fluidity, gene expression events, protein folding and degradation, assimilation of carbon sources, oxidative stress response, and production of specific amino acids and lipids culminate in *Listeria*'s successful persistence in the cold temperatures of the food processing environment (56).

An understanding of how *L. monocytogenes* proteome changes in the biofilm mode of growth at different temperatures can help to unveil how it establishes and survives in the processing environment. To this point, this study aimed to explore the *Listeria monocytogenes* biofilm subproteomes and surface proteins under the influence of three temperatures: 10°C mimicking a common setting found in the food industry environment, 25°C as a baseline temperature, and 37°C as the human host setting.

## Materials and Methods

### Strain and Biofilm Settings for Protein Extraction

The sequenced *L. monocytogenes* EGD-e strain, serogroup 1/2a, was used throughout this study (57). Routine pre-culturing and culturing were carried out in Tryptic Soy Broth (TSB, Difco, Fisher Scientific) at 25°C and 150 rpm. Bacterial growth was monitored by measuring the absorbance at 600 nm (OD_600_). Precultured cells in stationary phase were used to inoculate cultures to obtain a final OD_600_ of 0.005. After 6 h of growth, cells were harvested by centrifugation (7,500 × g, 15 min) and resuspended in TSB diluted by 1:5 with sterile water in a volume equal to that of the supernatant collected, reaching an OD_600_ between 0.6 and 0.7. Seven milliliters of the bacterial suspension was poured on each stainless steel (SS) disk (38.5 cm^2^), corresponding to an inoculation of 10^8^ to 10^9^ CFU/cm^2^ (colony-forming unit/ square centimeter). The SS disks were then placed in a sterile Petri dish (55-mm diameter) and incubated at 25°C. Bacterial cells were allowed to adhere onto the disk for 3 h in static mode, before removing the medium to eliminate planktonic cells and add fresh medium. The disks incubation settings were dependent on the desired temperature (10, 25, or 37°C) and growth stage (early stage or mature biofilm) (Supplementary Table 1). To harvest the biofilm, the medium was removed and adherent cells were detached in 10 ml of Tryptone-salt (tryptone 0.1%, NaCl 0.85%, pH 7.0) by scraping the SS disk with a sterile spatula. Three biological replicates with 10 disks per each were used for every analyzed setting. Cell adhesion and population in biofilm were evaluated by cell enumeration. Serial dilutions were plated on Tryptic Soy Agar (TSA, Difco, Fisher Scientific) and incubated for 24 h at 37°C.

### Protein Extraction Methods

The three extraction methods used in this study were biotin labeling, trypsin-enzymatic shaving and cell fractionation. All methods used were based on the protocol optimized for the extraction of surface exposed proteins developed by (58). In the biotinylation method, bacterial cells are treated with Sulfo-NHS-SS-Biotin, a marker molecule that is supposed to be membrane impermeable and interacts with surface exposed proteins. The shaving method consists of treating intact cells with proteases in an isotonic solution to release exposed peptides. The third method is the fairly well-established separation of membrane and cell wall components by cell fractionation. The technical details and result output of these extraction methods can be found in the Supporting information document and Supplementary Figures 1, 2.

### Nano-LC-MS/MS and Bioinformatic Analysis

In order to obtain triplicate protein extracts, three independent biofilm cultures were used for each of the three methods described above. All samples, except those from the shaving method, were loaded onto SDS-PAGE gels to concentrate in one single band in the first few millimeters of the resolution gel. Excised bands were washed in 25 mM ammonium bicarbonate with 5% acetonitrile (ACN) for 30 min and twice in 25 mM ammonium bicarbonate with 50% ACN for 30 min. Reduction and alkylation reactions were performed with 10 mM DTT and 55 mM iodoacetamide solutions, respectively, and all bands were finally dehydrated with 100% ACN. The samples were hydrolyzed overnight at 37°C using 48 μl of a 25 mM NH_4_HCO_3_/12.5 ng.μl^−1^ trypsin solution (Promega) per band. Peptides were extracted from the gel bands in an ultrasonic field during 10 min with 38.4 μl of 100% acetonitrile representing 80% of digestion volume. Supernatants were transferred in eppendorf vials and dried using Speed Vac for 45 min and 40 μl of equilibration solution (H_2_O/Trifluoroacetic Acid −99.95/0.05) was added.

All peptide mixtures were analyzed by nano-LC-MS/MS (Thermo Fisher Scientific) using an Ultimate 3000 system coupled to a LTQ Orbitrap Velos mass spectrometer (MS) with a nanoelectrospray ion source. For each sample, one microliter of peptide mixture was first preconcentrated on a C18 pre-column 5 cm length X 100 μm I.D. (Acclaim PepMap 100 C18, 5 μm, 100 A nanoViper), equilibrated with Trifluoroacetic Acid 0.05% in water at 30 μL/min. After 6 min of desalting and preconcentration, the pre-column was switched online with the analytical C18 column (75 μm inner diameter ×15 cm length; 2 μm, Acclaim PepMap 100 C18 Pepmap RSLC) equilibrated with 96 % solvent A (99.5 % H_2_O, 0.5 % formic acid) and 4 % solvent B (80%ACN, 19.5% H_2_O, 0.5% formic acid). Peptides were eluted according to their hydrophobicity at 300 nL/min flow rate using, respectively, a 4 to 52% gradient of solvent B for 31 min for biotin labeled and trypsin-enzymatic shaved fractions and a 4 to 40% gradient of solvent B for 56 min for cell fractions. Eluates were electrosprayed in positive-ion mode at 1.6 kV through a nanoelectrospray ion source heated to 250°C. The LTQ Orbitrap Velos MS was used in CID top 15 mode (i.e., 1 full scan MS and the 15 major peaks in the full scan were selected for MS/MS). The full scan MS was realized in the FTMS ion trap at a resolution of 60,000 (tolerance 10 ppm) and spectra were acquired with 1 microscan (m/z 400–1,400). For MS/MS, the following parameters were used: dynamic exclusion with 1 repeat counts, 20 s repeat duration and 80 s exclusion duration, isolation width for ion precursor was fixed at 1 m/z, single charged species were rejected, fragmentation used 37% normalized collision energy as the default activation of 0.25 and 10 ms activation time. For raw data processing, MS/MS ion search was performed with Mascot v2.5 for database search (http://www.matrixscience.com). The following parameters were considered for the searches: precursor mass tolerance of 10 ppm and fragment mass tolerance of 0.5 Da, a maximum of two missed cleavage sites of trypsin, carbamidomethylation (C) and oxidation (M) set as variable modifications. Protein identification was validated when at least two peptides originating from one protein showed statistically significant identity above Mascot scores 13 with a False Dicovery Rate of 1% (adjusted significance threshold *p* < 0.05). Ions score is −10 log(P), where *P* is the probability that the observed match is a random event. Individual ions score >13 indicate identity or extensive homology. Interrogations were performed against a custom database containing distinct entries corresponding to raw protein sequences and the different predicted mature proteins of *L. monocytogenes* EGD-e (i.e., DB-Mature-LmoEGDe v2.0, 5838 sequences) based on putative cleavage sites of the SP (59). The subcellular location of identified proteins was determined with the rational secretomics based strategy for genomic and proteomic analyses developed by Renier et al. (60). For protein quantitation analysis, LC-Progenesis was used with Mascot v2.3 and the same identification parameters described above. Normalization was based on the LC-Progenesis process with Log10 ratio calculation and scalar estimation in log space. The statistical method used was the comparison in groups of two temperature settings in a “Between-subject design.” For each temperature setting three biological replicas with three technical replicas were used. Venn diagrams were performed by the jvenn online tool (61). Functional category of proteins was based on the clusters of orthologous groups (COGs) via the eggNOG online framework (62). For proteins of interest, the average abundance from the 3 biological replicates in each condition was represented in a bar chart with standard deviation and statistical significance was tested by one-way ANOVA. Protein-protein association networks were made by the String database (63). Heat map displaying the normalized protein abundances were obtained via Xlstat (2018.5). Volcano plots were performed by the R software with the integration of the ggplot2 and ggrepel graphical packages for data analysis (64).

## Results

### Overall Protein Identification Results

**Figure 1:**
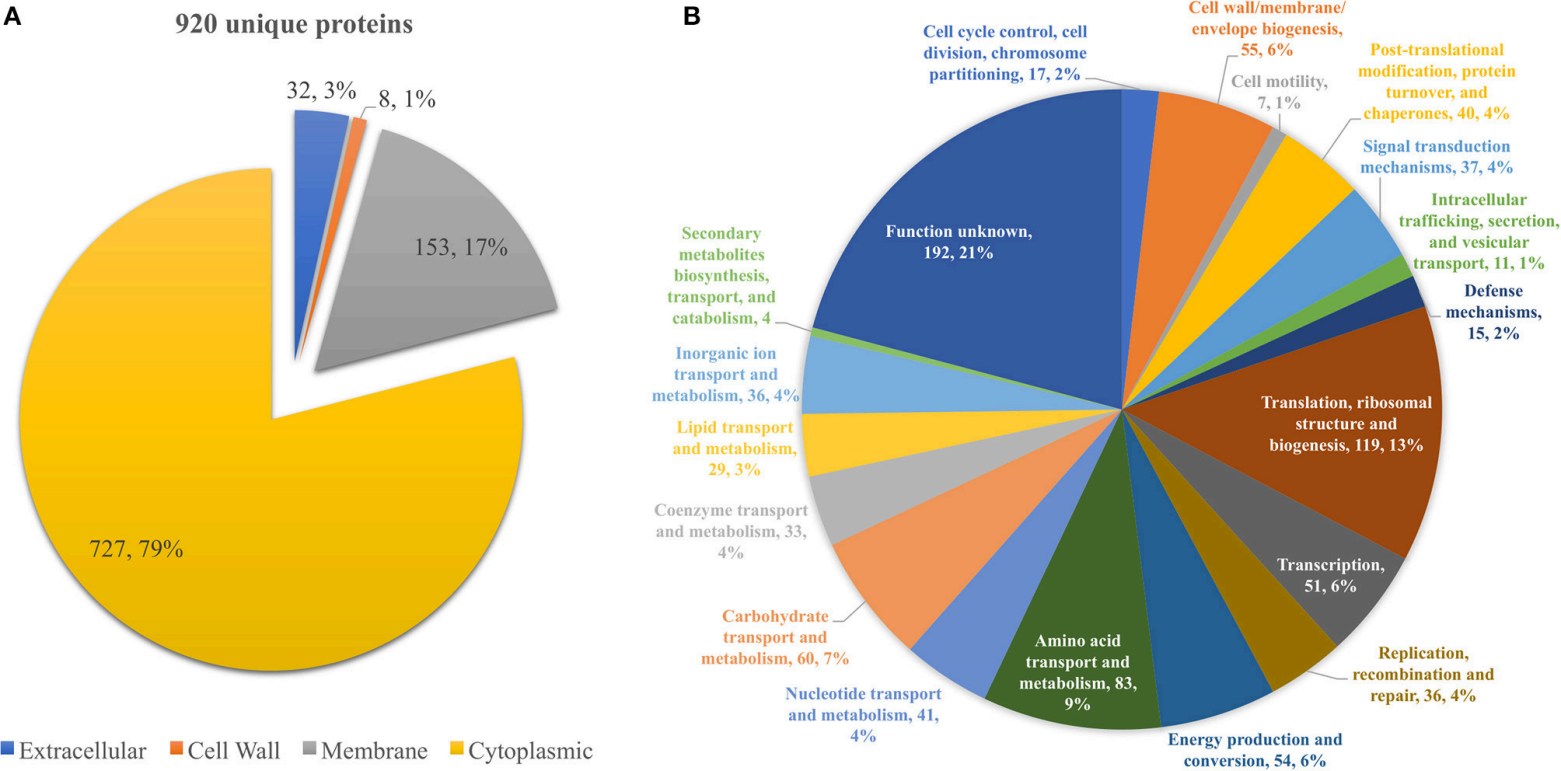
**(A)** Pie chart showing the distribution of the 920 unique proteins identified by their predicted subcellular localization. **(B)** Pie chart illustrating the distribution of the 920 unique proteins identified by their predicted COG molecular function.

**Figure 2:**
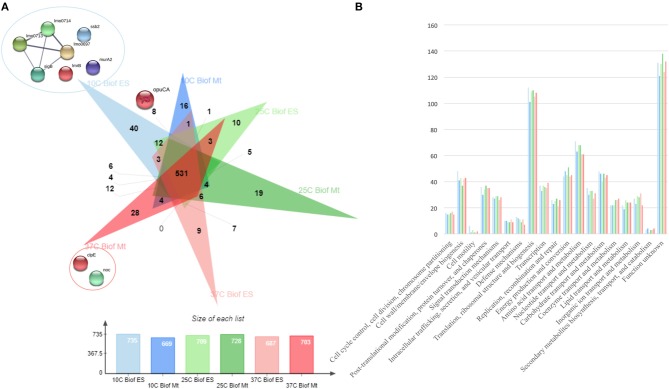
**(A)** Venn diagram exposing the distribution of the 920 unique proteins identified by the three sets of temperature (10, 25, and 37°C) and two growth stages (ES—Early Stage biofilm and Mt—Mature biofilm) here analyzed. **(B)** Vertical bar chart showing the distribution of the 920 unique proteins identified by their predicted COG molecular function and separated by the conditions in study.

From all extraction methods and temperature settings, 920 unique proteins were identified (Figure 1A), representing a significant proportion of the *Listeria monocytogenes* proteome (32.2%) (57). The vast majority, 79% of the identified proteins, were characterized as cytoproteins, followed by membrane-associated proteins, extracellular and cell wall-associated proteins represent 17, 3, and 1%, respectively. Functional category of this set of proteins was based on the clusters of orthologous groups (COGs) (Figure 1B). Taking into consideration that the majority of identified proteins are from the cytoplasmic subcellular localization, it is not surprising that the functional category with the highest percentage of identified proteins is “Translation, ribosomal structure, and biogenesis” (13%), followed by “Transcription” and ‘Cell wall/membrane/envelope biogenesis,” 6% each. In addition, there are a multitude of normal molecular functions with a similar number of identified proteins. Although the genome sequence of *L. monocytogenes* was performed 18 years ago, a significant part of its proteome is still unannotated and lacks relevant information. This is reinforced by the large number of proteins whose function is not known here (192, 21%). In regards to the subproteomic extraction approach here implemented, we identified a high percentage of the *L. monocytogenes* intracellular proteome and also a significant amount of its surfaceome. In order to look for potential protein biomarkers of temperature adaptation, the protein identification data was structured by temperature and growth condition. The column chart in Figure 4A illustrates the stable and steady amount of identified proteins that were found in the different settings. The six branched Venn diagram shows that the vast majority of proteins were shared between all settings (531 proteins). Nevertheless, 122 proteins were identified only in one of the six settings in the study. Some of them with potential interest with temperature adaptation. In the case of early stage biofilm (Biof Es) grown at 10°C (Figure 2A), 40 uniquely identified proteins were registered, including the RNA polymerase sigma factor SigB, documented as regulating the expression of genes necessary for survival under environmental stress conditions (65). We have also identified three proteins of the flagellar motility system (Lmo0713, Lmo0714, and Lmo0697) and a DNA replication, recombination and repair associated protein (ssb2). Interestingly, OpuCA which is part of a documented carnitine solute auto transporter was identified only at the 10°C settings. Likewise, a unique heat shock protein involved in cell division and virulence (clpE) was identified in the 37°C condition at mature biofilm growth (66). The distribution of molecular functions in each setting follows the same trend as in the analysis of the whole protein identification data set seen previously (Figure 2B). A higher amount of proteins related to translational machinery was present and a similar amount of proteins identified for each molecular function across the six different settings. Though, a comparison of protein flows and changes that might have a relation to temperature adaptation can only be achieved by quantitation analysis.

### Cross-Comparison of Temperature Adaptation Through Protein Quantitation

**Figure 3:**
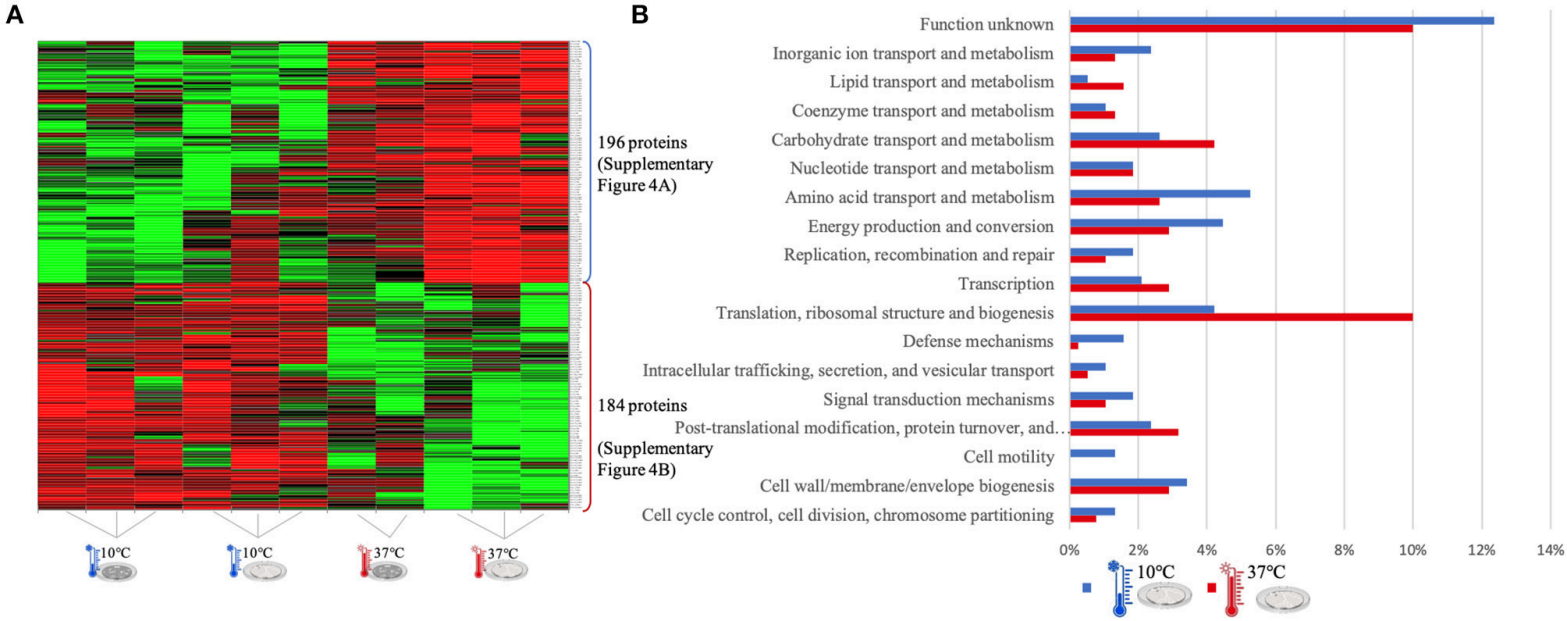
LC-Progenesis ANOVA comparing the biofilm at 10 and at 37°C (Supplementary Table 2) The two biofilm stages are represented either by dark SS (stainless still) disks for early stage or cell filled gray SS disks for mature biofilm. **(A)** Heat map illustrating the abundance of each of the 380 statistical significantly different proteins obtained in this comparison. **(B)** Horizontal bar chart showing the percentage of proteins with higher abundance in each temperature setting by their predicted COG molecular function.

**Figure 4:**
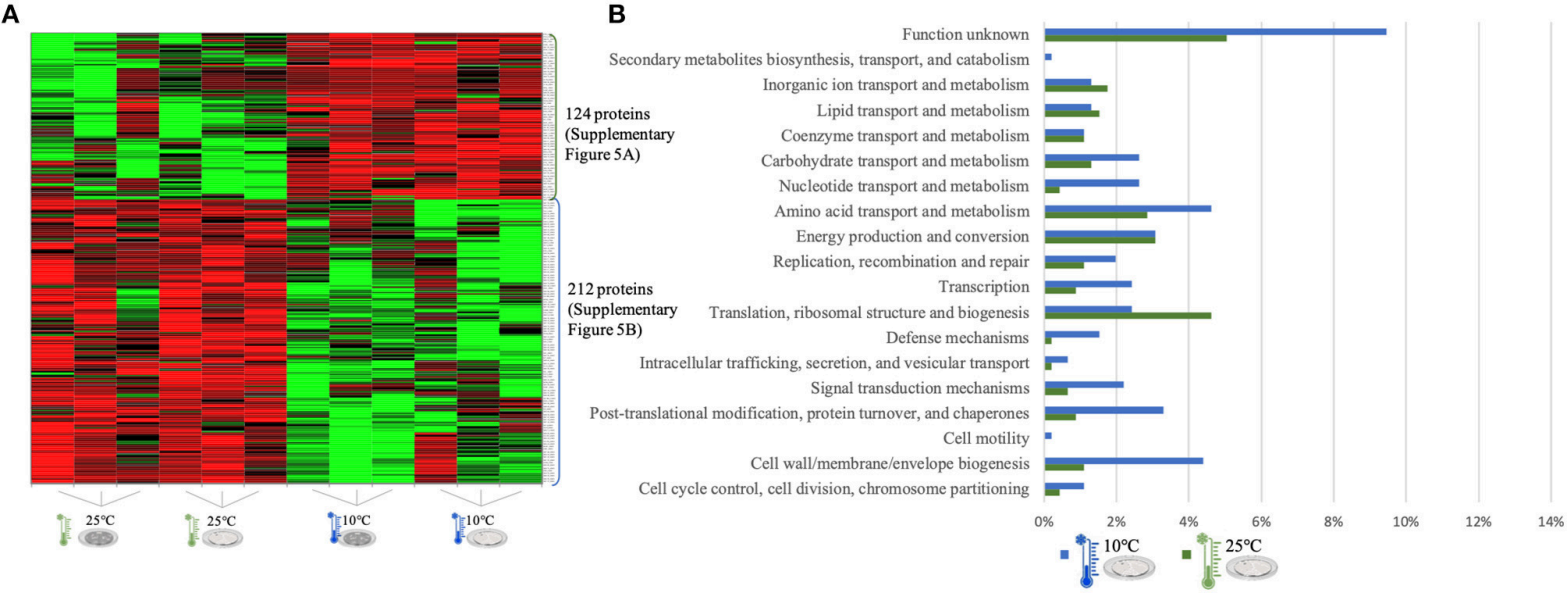
LC-Progenesis ANOVA comparing the biofilm at 10 and at 25°C (Supplementary Table 3). The two biofilm stages are represented either by dark SS (stainless still) disks for early stage or cell filled gray SS disks for mature biofilm. **(A)** Heat map illustrating the abundance of each of the 336 statistical significantly different proteins obtained in this comparison. **(B)** Horizontal bar chart showing the percentage of proteins with higher abundance in each temperature setting by their predicted COG molecular function.

**Figure 5:**
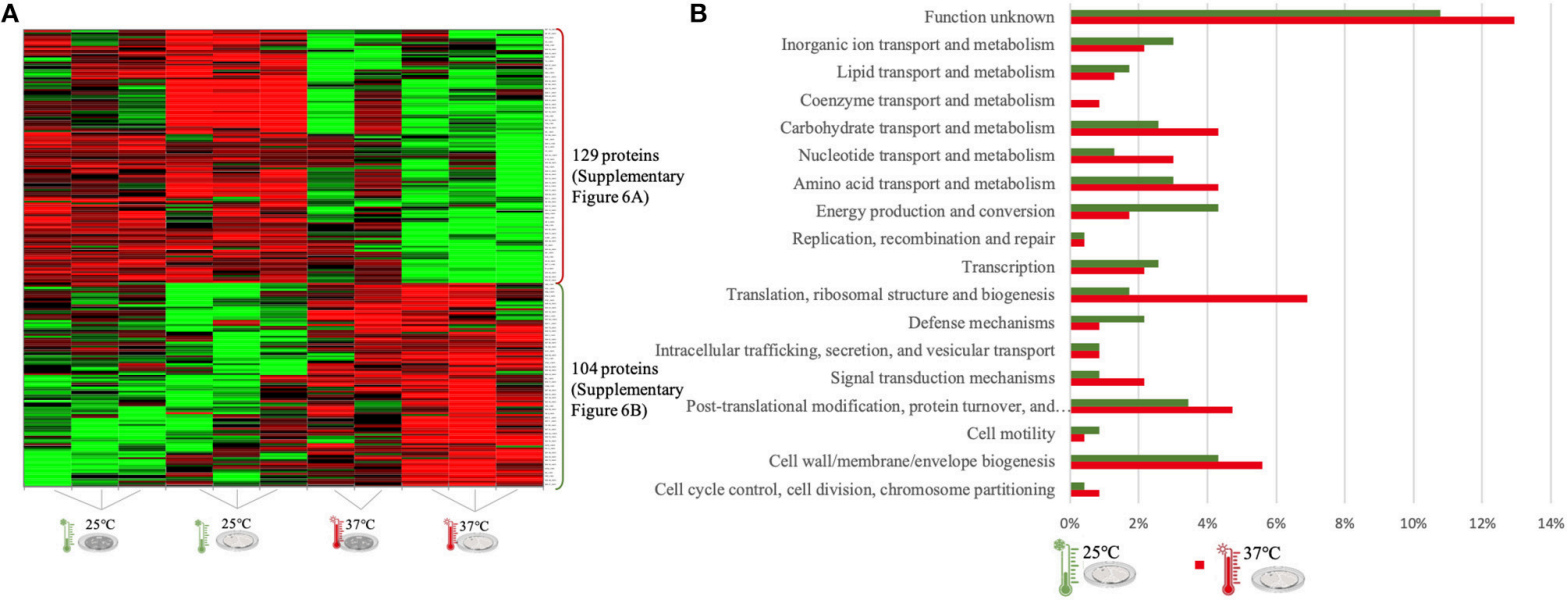
LC-Progenesis ANOVA comparing the biofilm at 25 and at 37°C (Supplementary Table 4). The two biofilm stages are represented either by dark SS (stainless still) disks for early stage or cell filled gray SS disks for mature biofilm. **(A)** Heat map illustrating the abundance of each of the 233 statistical significantly different proteins obtained in this comparison. **(B)** Horizontal bar chart showing the percentage of proteins with higher abundance in each temperature setting by their predicted COG molecular function.

The quantitative analysis of protein abundance made by LC-Progenesis enabled the comparison of protein abundance through pairs of temperature settings, 10 vs. 37°C (Figure 3; Supplementary Figure 4 and Supplementary Table 2), 10 vs. 25°C (Figure 4; Supplementary Figure 5 and Supplementary Table 3), and 25 vs. 37°C (Figure 5; Supplementary Figure 6 and Supplementary Table 4). In all cases, LC-Progenesis normalized abundances of the statistically significant identified proteins (*p* < 0.05) were used in order to compare the flows of proteins in each setting.

The Xlstat heat map in Figure 3A shows the relative abundance of each of the 380 statistically significant proteins retrieved when comparing the two growth phases at 10 and 37°C. This was the most contrasting comparison of growth temperatures, between a temperature mimicking the settings found in the food industry (10°C) and the temperature of the human host (37°C) to which *L. monocytogenes* expresses a higher level of virulence. A similar sum of proteins with higher abundance was detected in both temperatures, i.e., 196 at 10°C and 184 at 37°C. In some instances, there is a variance in one or more biological replicates regarding its abundance. The comparison of proteome changes can also be viewed by the shifts of protein abundance in regards to molecular function (Figure 3B). Regarding to transcriptional or translational activity, at 37°C, there are more proteins with higher abundance annotated with this molecular function. On the other hand, a higher percentage of proteins associated with a repair system mechanism were more abundant in the cold condition than at 37°C. The repair system molecular function is one of the categories COG (EggNOG orthologs). A temperature stress as 10°C induces DNA, RNA and protein damage in the cell (45). Some of the proteins that were more abundant at 10°C in this category have annotated involvement in solving protein aggregation and DNA damage (as for example DNAA chaperone protein, Supplementary Table 2). Similar result was seen for inorganic ion and amino acid transport associated proteins. A String map of protein interaction (Supplementary Figure 4) provides a graphical representation of the statistically significant proteins from this quantitation. Highlighted in blue are some of the proteins associated with the adaptation to harsh environmental conditions that were more abundant in the 10°C condition (Supplementary Figure 4A), as is the case of: the GTP-sensing transcriptional pleiotropic repressor CodY; the carnitine transporter OpuCA; the redox regulated molecular chaperone HslO, that protects both thermally unfolding and oxidatively damaged proteins from irreversible aggregation (67); the foldase protein PrsA2, which protects the cell by controlling the folding and stability of secreted proteins in stress conditions (68); the DNA protection during starvation Dps, that protects DNA from oxidative damage by sequestering intracellular Fe^2+^ ion and storing it in the form of Fe^3+^ oxyhydroxide mineral (56); the ATP-dependent zinc metalloprotease FtsH, which plays a role in the quality control of integral membrane proteins (69); the DNA ligase LigA, essential for DNA replication and repair of damaged DNA under stress conditions (70); the superoxide dismutase SodA, responsible for destroying superoxide anion radicals which are produced within the cells (71); and the ATP-dependent RNA helicases CshA and CshB, which are involved in RNA degradation during cold tolerance, motility and alcohol tolerance (72).

The large sum of ribosomal proteins at 37°C validates the higher percentage of proteins previously associated with translational function (Supplementary Figure 4B). Likewise, highlighted in red are some of the chaperone proteins associated with heat adaptation and the general stress response system, that were more abundant at 37°C. They participate actively in the response to hyperosmotic and heat shock by the recognition and processing of DNA lesions, and preventing the aggregation of stress-denatured proteins and by disaggregating proteins (73), such proteins as: DnaK, DnaJ, GroES, GroEL, GrpE, UvrA, and ClpB. The virulence capacity of *L. monocytogenes* is also here underlined by the higher abundance of the endopeptidase p60 (Iap) which is a major extracellular protein involved in the invasion of phagocytic cells (11).

The quantitation appraisal of the cold condition to the control temperature setting in this study (25°C) resulted in 336 statistically significant proteins (Figure 4A). We observed 26% more proteins with a higher abundance at 10°C than at 25°C. In agreement with the previous comparison, sessile cells at 10°C showed a lower percentage of abundance of proteins associated with translational machinery and, once again, a higher percentage of proteins with higher abundance involved in amino acid transport. The mapping of this set of proteins reveals the same trend as previous results with stress response associated proteins being more abundant in the cold condition (e.g., ClspL and SigB) (Supplementary Figure 5). The higher number of ribosomal proteins at 25°C confirms the tendency toward fewer translation mechanisms at the cold condition. Furthermore, chaperone proteins (DnaK, GroEL) and some virulence associated proteins, like the internalin A (InlA) which mediates the entry of *L. monocytogenes* into host intestinal epithelial cells (74) and chemotaxis CheA involved in the transmission of sensory signals from the chemoreceptors to the flagellar motors in intracellular movement (75), were also present in lower levels at the colder setting.

The last quantitative comparison of protein abundance was between 25 and 37°C, which gave the lowest amount of statistically significant proteins among the three comparisons (233 proteins, Figure 5A). A similar number of proteins with higher abundance was found under both conditions, probably related to the proximity of this two temperature settings. However, changes in proteome abundance can be retrieved upon analysis of molecular functional categories (Figure 5B). As in the previous two analyses, the highest temperature showed a greater number of proteins associated with translation. At 37°C, there is a higher amount of proteins with higher abundance related to carbohydrate metabolism and also coenzyme transport. The flagella motor system was more abundant at 25°C (Supplementary Figure 6A). As in the first quantitative appraisal (10 vs. 37°C), the ribosomal, chaperone and virulence associated proteins were more abundant at 37°C (Supplementary Figure 4B).

### Proteins of Interest for Temperature Adaptation

**Figure 6:**
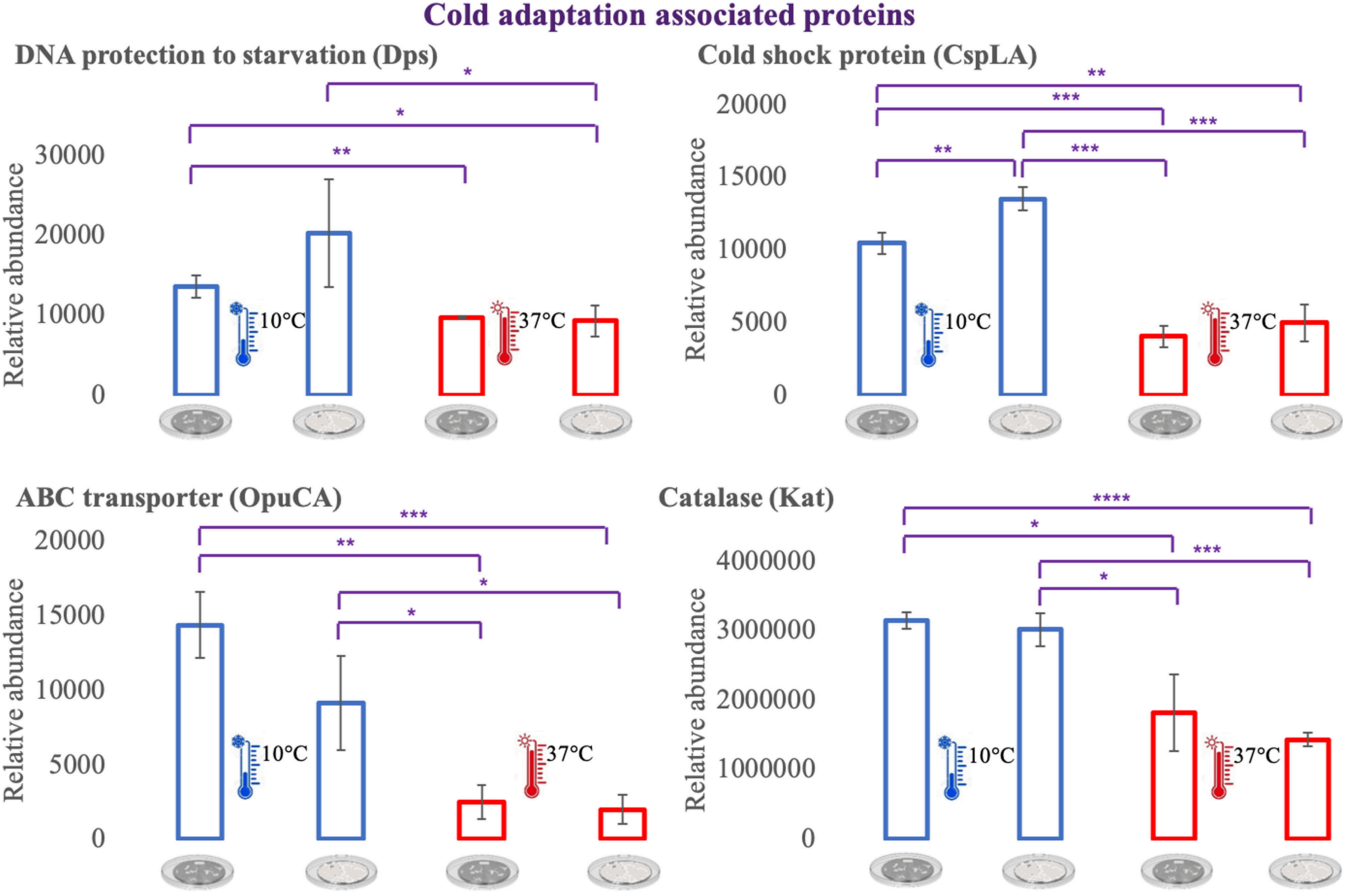
Vertical bar charts showing the average abundance of some cold adaptation associated proteins by the different condition in analysis. Statistical significance was tested by one-way ANOVA (^*^P ≤ 0.05; ^**^P ≤ 0.01; ^***^P ≤ 0.00; ^****^P ≤ 0.0001). The two biofilm stages are represented either by dark SS (stainless still) disks for early stage or cell filled gray SS disks for mature biofilm.

**Figure 7:**
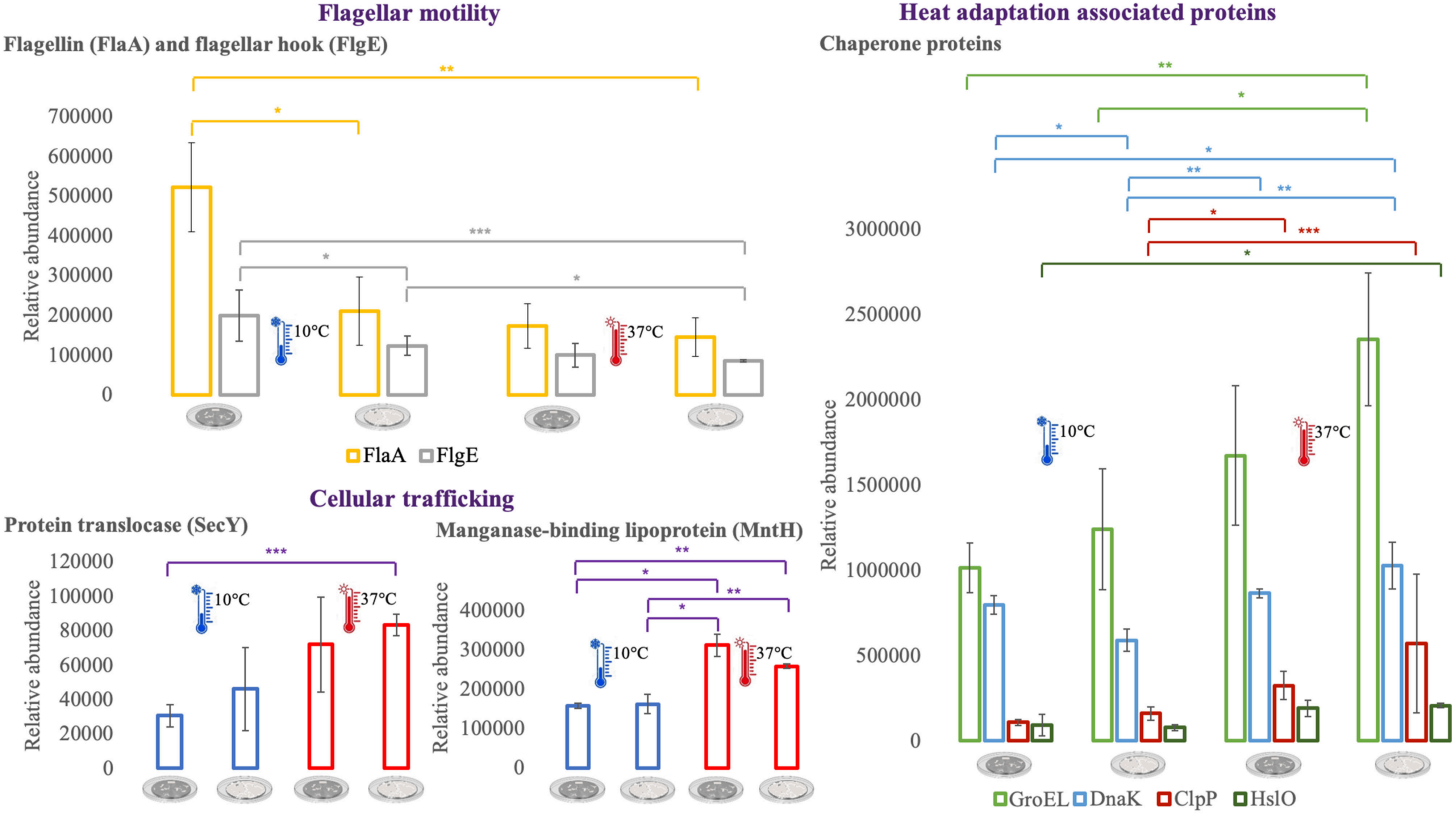
Vertical bar charts showing the average abundance of flagellar, cellular trafficking and heat adaptation associated proteins by the different condition in analysis. Statistical significance was tested by one-way ANOVA (^*^P ≤ 0.05; ^**^P ≤ 0.01; ^***^P ≤ 0.00). The two biofilm stages are represented either by dark SS (stainless still) disks for early stage or cell filled gray SS disks for mature biofilm.

**Figure 8:**
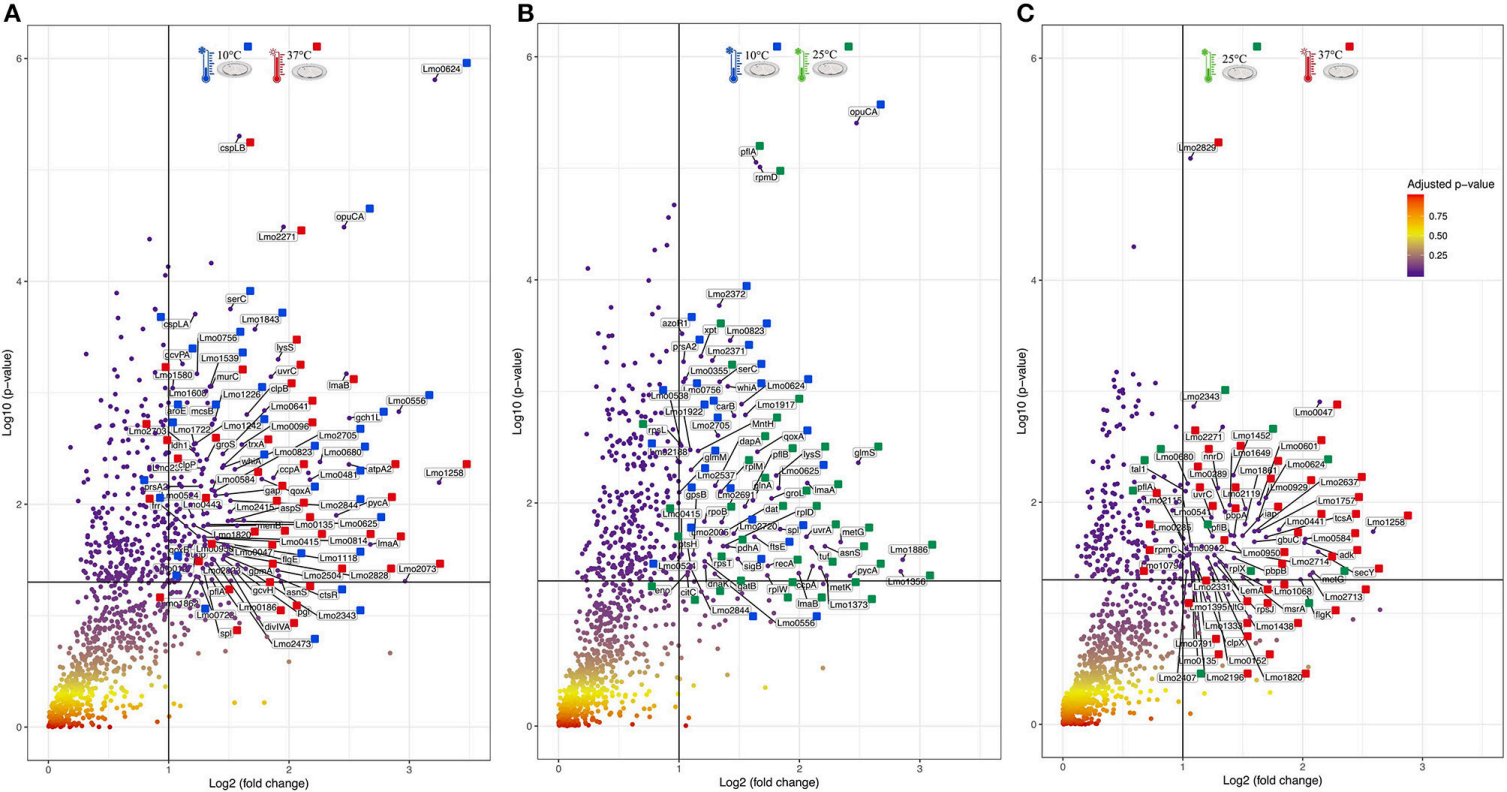
Volcano plots representing the distribution of the identified proteins taking into account their fold change and *p*-value, Cut-off are represented by black lines at fold change two [Log_2_(fold change) = 1] and *p*-value 0,05 [Log_10_(*p*-value) = 1.30103]. The top 50 proteins with highest fold change are marked with their respective color that represent in which setting they were more abundant (blue for 10°C, green for 25°C, and red for 37°C). **(A)** Volcano plot for the comparison between 10 and 37°C. **(B)** Volcano plot for the comparison between 10 and 25°C. **(C)** Volcano plot for the comparison between 25 and 37°C.

For an additional understanding of the *L. monocytogenes* proteome changes under different conditions, it was important to have a look at sets of proteins of interest and also outliers that could be potential biomarkers for the adaptation to this environmental and food industry setting. Figures 6, 7 represent the average abundances of some of these proteins of interest between the two extreme poles of temperatures and across the two stages of biofilm growth (early stage, represented by dark SS disks, and mature biofilm, represented by cell filled SS disks). For each protein, average abundances were obtained from the three biological replicates in each condition. In the case of the DNA protection to starvation protein (Dps), a higher abundance was obtained at 10°C, particularly in the mature biofilm stage. Dps is important for full resistance to heat and cold shocks and is essential for full virulence of this bacterium (56). The abundance of other three cold adaptation associated proteins was also greater at 10°C than at 37°C (Figure 6), namely: the cold shock protein (CspLA); ABC transporter OpuCA; and Kat, a catalase suggested to be one of the contributors to the ability of *L. monocytogenes* to grow at low temperatures (76). The cell motility associated proteins were more abundant at temperatures under 37°C, as it is the case of flagellin (FlaA) and flagellar hook (FlgE) with a higher abundance at 10°C, particularly in the early stage biofilm (Figure 7). A different tendency was seen for the cellular trafficking proteins, the abundance of these proteins were registered with higher levels at 37°C. Heat adaptation associated protein chaperones were detected, in most instances, with higher abundance at 37°C, particularly in the mature biofilm. To further retrieve protein drifts and outliers, volcano plots were generated from the three groups of temperature comparison, with cut-offs at *p*-value = 0.05 (log10(*p*-value) = 1.30103) and fold change (FC) = 2 (log2(fold change) = 1). Each of the quantitative comparisons was represented in a different volcano plot, 10 vs. 37°C (Figure 8A), 10 vs. 25°C (Figure 8B), and 25 vs. 37°C (Figure 8C). A total of 139, 68, and 58 proteins respected these cut-offs criteria, respectively. In all three plots, the top 50 proteins with higher fold change were marked with the color to which they presented higher mean condition (blue for 10°C, green for 25°C, and red for 37°C). In the 10 vs. 37°C plot, the proteins with the highest fold change at 10°C are associated with metabolism and amino acid transport. In the 10°C highlighted set of proteins are also included stress adaptation proteins, such as OpuCA (Q7AP65_LISMO) and CspLA (CSPA_LISMO), and cell motility associated proteins (Q8Y954_LISMO, Lmo0680). In the pool of proteins with high fold change at 37°C, there are proteins associated with virulence as LmaA (Q7AP93_LISMO), LmaB (Q7AP94_LISMO), CspLB (CSPB_LISMO) and proteins involved in heat adaptation (Lmo1580, Q8Y6V1_LISMO, and CcpA, Q8Y6T3_LISMO). In regards to the evaluation between 10 and 25°C, transport associated proteins were observed with the highest fold change in the cold condition. This was particularly the case for amino acid transporter such as multiple ATP-binding cassette transporter (ABC) OpuCA, (Q7AP65_LISMO), Lmo0538 (Q8Y9J0_LISMO), and SerC (SERC_LISMO). At 25°C, most of proteins had a connection with energy production and translation machinery. At the level of the 25 vs. 37°C comparison, the majority of proteins were associated with translation or carbohydrate functions. To note the presence of two virulence associated proteins with FC>2 at 37°C, Iap (P60_LISMO) and UvrC (UVRC_LISMO).

## Discussion

*Listeria monocytogenes* is the causative agent of listeriosis, a highly fatal disease to fetuses, newborns, infants, pregnant women, elderly and immunocompromised individuals (77). Invasive listeriosis is considered the leading cause of death from foodborne infections in industrialized countries. Despite the application of the food safety criteria (FSC) for *L. monocytogenes* in RTE foods from 2006 onwards, a statistically significant increase in listeriosis cases has been reported in the European Union between 2009 and 2013 (3, 78).

The cleaning and disinfection of surfaces and materials in food industries is difficult to achieve due to *L. monocytogenes* capacity to survive under harsh condition, such as a wide pH range (79), desiccated environments (9, 20), and low temperatures (80). Since *L. monocytogenes* is a psychrotrophic microorganism able to multiply in food stored under refrigerated temperatures, a low contamination number present in RTE products can grow to a level which threatens consumer health (81). At low temperatures, *Listeria* faces multiple molecular constraints such as increased membrane rigidity, amino acid starvation, oxidative stress, aberrant protein synthesis, cell surface remodeling, reduced protein and enzyme activity, slow transport, and nutrient uptake processes (56, 82). *L. monocytogenes* response mechanisms to these hurdles has been extensively studied by transcriptomic approaches. Contrasting are the few studies to the changes at the proteome level that allow *Listeria* to survive at refrigeration temperatures (41, 43, 83), and none performed in a biofilm mode of growth. The present study sought to investigate the influence of three temperatures in the proteome changes of *L. monocytogenes* grown in the biofilm mode of growth. In the next subsections, we overview some noteworthy protein abundance shifts associated with the major adaptation to cold mechanisms present in *L. monocytogenes*, namely: the role of ribosomes and the stressosome, changes in cell fluidity and motility, the uptake of osmolytes, and the relevance of the overexpression of chaperone proteins such as cold shock proteins. Based on the proteomic data obtained in this study it is highly unlikely that a set of surface proteins play a unique role in the adaptation to the different temperatures. Hence, adaptation to low temperature growth is a complex response involving many aspects of the cell molecular biology and biochemistry (45). The typical molecular mechanisms of cold stress adaptation referenced in bacteria include: overexpression of stability mechanisms by the modulation of nucleic acid structures, maintenance of structural integrity in cell membranes, uptake of compatible solutes, production of various cold stress proteins, including cold shock proteins (Csps), and nonspecific stress response mechanisms.

### Cold Stress Adaptation and the Role of Ribosomes

One of the first side effects of exposure to low temperatures is the compromise of ribosomal structural stability and this deterioration causes a general reduction in the bacteria's protein synthesis capacity (45). In this study, the majority of ribosomal proteins were less abundant in the cold condition, and in some instances, ribosomal proteins had a high fold change in the warmer setting (Figure 8). The 50S ribosomal proteins (Rl) are referenced as the first cold stress sensors in microbes, as is the case of Rl11. The former is essential for the activation of sigma B (σ^B^) transcription factor in *Bacillus subitilis* (84), the general stress response transcriptional factor that controls the expression of dozens of stress adaptation related genes. We observed an increased level of Rl11 protein at 10°C, as well as the initiation factor IF2 which is implicated in the formation of 30S preinitiation complex, suggesting a role in ribosome assembling (41). The general stress protein Ctc (Rl25), was here more abundant at 10°C and the same was seen in *L. monocytogenes* upon exposure to a salt condition (85). Concerning σ^B^, this general stress transcription factor was more abundant at 10°C than at the room temperature (Supplementary Table 3). The σ^B^ enables *Listeria* pathogenicity in two different ways. First, by controlling the expression of genes that enhance the survival during food industry pipeline settings. Second, σ^B^ plays a role upon infection by indirectly regulating PrfA, the main virulence regulator (86, 87). This transcriptional factor has also been associated with the efficient accumulation of betaine and carnitine as cryoprotectants (88), which will be further discussed in the next sections. On the contrary, it has been suggested that σ^B^ does not, in fact, play a pivotal role during cold adaptation in *L. monocytogenes*. So, it seems to contribute to the adaptation in a growth phase-dependent manner, particularly in the early stages of growth (89). This result was not seen here, since σ^B^ was more abundant in the mature biofilm than in the early stage one. The reasoning behind this can be linked with the different modes of growth applied in the two studies and the fact that σ^B^ also has a determinant role in biofilm formation (90).

### Cell Membrane Fluidity, Chemotaxis, and Their Involvement in Cold Adaptation

Changes in the cell membrane fluidity and surface proteins are part of the bacterial adaptation to cold stress (30). For example, Lmo0624 has a role in lipid metabolism and higher transcripts of this gene were reported at 4°C (91). In agreement with this result we also observed Lmo0624 (Q8Y9A8) more abundant in the cold condition with one of the highest fold changes (FC = 9.27) (Figure 8A). The foldase protein PrsA2, here found more abundant in the colder setting (FC = 2.41, Supplementary Table 2) is a member of a family of membrane-associated lipoproteins that play a role in the folding and stability of secreted proteins as they pass the bacterial membrane. PrsA2 contributes to the integrity of the *L. monocytogenes* cell wall as well as swimming motility and bacterial resistance to different stresses (92). Cell stability and bacterial attachment to surfaces are influenced not only by cell surface properties but also by the presence of surface appendages, such as flagella (93). All the cell motility associated proteins identified in this study, namely flagellin (FlaA), flagellar hooks FlgK and FlgE, Lmo0689 (Q8Y948), Flha (Lmo0680, Q8Y954), MotA (Lmo0685, Q7AP82) were more abundant at 10 and 25°C when compared with the *in vivo* setting (37°C). FlaA is the bacterial flagella main protein (94) and *L. monocytogenes* strains are motile and flagellated below 30°C (95), and typically not motile at 37°C (96). However, the role of the flagellum in biofilm formation is controversial. There are observations that give it an important role in biofilm formation (97), and in other reports strains with this gene deleted had improved sessile development (98). In regards to the putative role of the flagella, it was described to be required for the initial cell attachment phase by overcoming the van der Waals forces (93). We have observed several motility associated proteins with significant abundance shifts that support the hypothesis that motile flagellum is needed for an optimal cold stress response in *L. monocytogenes*. Moreover, we have detected that all these cell motility proteins were more abundant in the early stage of biofilm. A flagellum-associated operon (consisting of Lmo0675 to Lmo0689) has been previously retrieved in high transcript levels from *L. monocytogenes* exposed to 4°C in log phase (91). Lmo0689 was also observed in this study to be more abundant at 10°C and in the early stage biofilm. Similarly, FlhA and MotA play a role in the cold tolerance of *L. monocytogenes* (99). This is consistent with our observation that MotA was one of the proteins with the highest fold change (FC = 4.78) in the 10 vs. 37°C comparison. Likewise, the ability to modulate membrane fatty acid composition and improve membrane fluidity is a crucial step for temperature adaptation (100). FabH, which was here more abundant at 10°C, is connected with the increased formation of anteiso branched-chain fatty acids during cold temperature adaptation, which ultimately is responsible for the increase in membrane fluidity (101).

### The Crucial Role of Cryoprotective Solutes

The uptake of compatible solutes/osmolytes is one of the key steps for the survival of bacteria in stress conditions. Osmolytes are low-molecular weight organic compounds that are stored in high intracellular concentrations with minimal effects on the normal functioning of the cell. At low temperatures, cryoprotective solutes act through stabilization of enzymatic functions and the cell membrane lipid bilayer (102). Organic compounds such as glycine betaine and carnitine help to relieve turgor pressure, in this manner they have an essential role in the survival of *Listeria* under elevated osmolarity and cold settings (25, 50). In the food industry, *L. monocytogenes* has access to these solutes from carnitine rich meat and dairy products, and glycine betaine from plants and shellfish (54). There are three solute import systems known to operate in *L. monocytogenes*: glycine betaine porter I (BetL), glycine betaine porter II (Gbu), and the carnitine transporter OpuC (25). Here, we have identified GbuB (Q7AP75) and OpuCA (Q7AP65). The non-identification of a BetL can be reasoned with the described more pronounced role of Gbu and OpuC as preferential cryoprotection systems in *L. monocytogenes* (49). Regarding the cryoprotective impact between Gbu and OpuC, a report comparing their role concluded that carnitine uptake at low temperatures is higher than betaine after cold temperature (48). Supporting this result, GbuB (Q7AP75) and OpuCA (Q7AP65) were in this study more abundant at 10°C. Interestingly, OpuCA had one of the highest fold changes (FC = 4.91, Figure 8A; FC = 5.56, Figure 8B), one more hint that cryoprotectants are essential for *Listeria* rapid response to the conditions typically found in food preservation.

### Cold Shock Proteins and Other Proteins Potential Involved in Temperature Adaptation

A second key point into the adaptation of a microbe to a cold stress is the production of various stress-related proteins, including cold shock proteins (Csps) (31). Our results showed a higher abundance of the cold shock protein CspLA (CSPA) at 10°C and with high fold change (FC = 2.33, Figure 8A). CspA deletion mutants have showed to abolish *L. monocytogenes* growth at refrigeration temperatures (31). However, there are also Csps downregulated at low temperature, pointing to their potential role under conditions other than cold growth (91). Similar result was here obtained with the cold shock protein CspLB (CSPB) more abundant in the warmer condition (FC = 3, Figure 8A). This infers that CspLB also have a function during normal growth (40).

Various other cold response associated proteins were identified in this study. The GTP-sensing transcriptional pleiotropic repressor CodY was more abundant at 10°C. CodY has a role in the regulation of relevant genes implicated in *Listeria* growth at low temperature (91). *L. monocytogenes* CodY also has a recognized role in virulence (65), in this matter, from the 16 proteins identified here encoded by CodY regulated genes there are cases of stress adaptation associated proteins (Dps and SerC—more abundant at 10°C) and virulence-associated proteins (LmaA, LmaB, GroES, GroEL, and ClpB—more abundant at 37°C) (Supplementary Table 2). DNA protection during starvation protein (Dps or Fri) is a 18 kDa *L. monocytogenes* major cold shock protein that is required for iron storage and protection against reactive oxygen species (55, 103). This major protein for low-temperature adaptation was here detected in high levels at 10°C. Similar result was obtained for enolase (Eno) (Supplementary Table 2), which has been found to be upregulated at low temperatures by others (43). Eno does not possess an N-terminal signal peptide, but it was found before to be exposed on the bacterial cell surface or extracellularly, thus meaning there should be an uncharacterized secretion pathway (94). Further proteins connected to cold adaptation mechanisms in *L. monocytogenes* were more abundant at 10°C, such as folding catalysts Lmo1583 and Lmo2376, that have been detected before in increased levels at 4°C (41). Proteins with a role in protection from reactive oxygen species (superoxide dismutase-Sod, catalase-Cat, Lmo0640, and Lmo1967) and in iron metabolism (Lmo2415-SufD and Lmo2411-SufB) were also more abundant in the cold condition. SufD and SufB are part of the Suf system, which in *Escherichia coli* is activated to enable the increase of *de novo* Fe–S assembly, maintaining Fe–S cluster biosynthesis under oxidative stress conditions (104). Two-component-system histidine kinases (TCSs) are among the major systems that aid bacteria in overcoming many of arduous stress factors encountered in nature and during food processing environment (28). LisK which is part of the TCSs was here more abundant at 10°C. As for RNA chaperones, like DEAD-box RNA helicases, they act by resolving secondary structures in mRNA that can be developed in a cell subjected to cold (105). The three putative DEAD-box RNA helicase genes (Lmo0866, Lmo1450, and Lmo1722) are required for cold tolerance and motility in *L. monocytogenes* (37, 106). We have observed the three helicases with higher abundances at 10°C. The overall protein abundance data obtained also showed some nonspecific stress response mechanisms or shared responses to other stress conditions. Such a case is the amino acid, lipid and carbohydrate transport which have at least a double role in the protection against cold temperatures and salt exposure (83). In respect to the proteins associated with the amino acid transport and metabolism, the majority of them was more abundant in the cold condition (66.7% in 10 vs. 37°C, Figure 3B; 61.8% 10 vs. 25°C, Figure 4B). This suggests that the cells endure starvation in certain amino acids during a cold condition, counterbalancing this lack with the increase expression of such transportation systems. Moreover, some amino acid biosynthetic enzymes were also more abundant at 10°C, for example Cysteine tRNA ligase (CysS) and Shikimate dehydrogenase (AroE, Q8Y733), indicating that the stressed cell responds by increasing the production of enzymes needed to upturn the production of scarce amino acids (45, 82). Additionally, six proteins with amino acid transport presented a high fold change, including AroE with FC = 2.23.

### Abundance Flows of Virulence-Associated Proteins

The comparison made between a food processing low temperature setting and the *in vivo* temperature enabled an outlook over the changes in heat shock proteins (HSP) and proteins typically associated with virulence. HSPs main function is to repair damage to proteins via chaperone activity, for example DnaK, GroEL, GroES, GrpE, ClpB, and HtpG (27). One might assume their importance in resolving protein damage from cold stress, however this class of stress response genes are usually less transcribed at lower temperatures (91). In this study, the majority of these chaperone proteins were more abundant in the warmer condition, particularly at 37°C. This is also connected to the fact that *L. monocytogenes* is a psychrotroph microorganism and growth in temperatures above 35°C may result in the induction of a stress response (107). Moreover, chaperone proteins are also required for *L. monocytogenes* maximum virulence potential (82). To approach the topic of virulence-associated proteins, it is pertinent to look into Lmo0443, a potential factor for low virulence. Lmo0443 is overexpressed in less virulent *L. monocytogenes* strains and underexpressed in more virulent ones (108). In this *L. monocytogenes* EGD-e strain, Lmo0443 had higher abundance at 37°C, consistent with its lower pathogenicity when compared with EGD or 10403S *Listeria* strains (109). In the same trend of results, internalins are essential for *Listeria* pathogenicity (110). Only one internalin was detected in this study. InlA which was observed with higher abundance at 25°C (10 vs. 25°C comparison, Supplementary Table 3). Internalins have a diversity of functions amongst the Listeriae, InlA in particular is documented as being crucial for *Listeria* capacity to invade human epithelial cell lines (111). The crucial topological factor (DivIVA) required for completion of cell division in *L. monocytogenes* and associated with host cell invasion was more abundant in the 37°C condition with high fold change (FC = 2.84) (13). Lastly, the *agr* locus of *L. monocytogenes* is recognized to be important in bacterial virulence. The open reading frame *lmo0047* that proceeds the *agrB* encodes a protein of unknown function (112), that was here more abundant at 37°C and with high fold change (FC = 4.41), suggesting a potential role in a virulence mechanism.

Consistent with previous studies (30, 32, 35, 37, 38, 41, 43, 44, 56, 82, 83, 85, 91), we have observed a significant remodeling of protein abundance as a function of temperature, highlighting once again the predominant influence of this environmental parameter on protein expression and the need for the bacteria to adapt to it and maintain its homeostasis.

## Conclusion

*Listeria monocytogenes* overcomes various kinds of stress, including the low temperatures present in food processing and storage. Cold stress adaptation mechanisms are therefore an essential skill of *Listeria*, enabling it to survive and proliferate to reach minimal infectious levels on refrigerated foods. In this aspect, the cold conditions in food plants may, in fact, be selecting for *L. monocytogenes* subtypes with the appropriate adaptive physiological attributes that lead to efficient survival and spread during food handling (45). Analogous approaches in the future will aid in highlighting additional potential target genes of cold stress resistance in *Listeria monocytogenes*. It will be of particular interest to make a direct comparison between this biofilm proteomic data and similar planktonic approaches. Thus, enabling the study of possible molecular cold response and biofilm targets that currently await additional assessment.

## Author Contributions

TS and MH designed the study. TS wrote the manuscript. TS, CC, DV, and JE performed the experiments. TS, DV, and MH analyzed the data. All authors reviewed the manuscript.

### Conflict of Interest Statement

The authors declare that the research was conducted in the absence of any commercial or financial relationships that could be construed as a potential conflict of interest.
