# Supplementary material for: Listeria monocytogenes Biofilm Adaptation to Different Temperatures Seen Through Shotgun Proteomics
Source: Front Nutr. 2019 Jun 14;6:89. doi: 10.3389/fnut.2019.00089 (PMC6587611; doi:10.3389/fnut.2019.00089)
Supplement: Supplementary file 5 [file Data_Sheet_1.PDF]

Supporting information

Protein extraction methods

The biotinylation method uses a cell-impermeable, cleavable Sulfo-NHS-SS-Biotin to label exposed primary amines of proteins on the surface of intact cells. Biofilm cells were suspended in 10 ml buffer A (PBS, 0.01 mM, pH 8 + 1 mM PMSF). The suspension was transferred into weighted tubes and centrifuged at 4,000 x g for 10 min at room temperature (RT). The bacterial pellet was washed twice, and the weight of wet cells was calculated. Each 100 mg of cells was resuspended in 300 µl buffer A supplemented with 1.5 mM EZ-Link Sulfo-NHS-SS (Thermo Scientific). Biotinylation was performed for 15 min at RT under gentle agitation. Free biotin was removed by centrifugation at 4,000 x g for 5 min at RT and washing the pellet three times with PBS (0.01 M, pH 8 + 500 mM glycine). Cells were resuspended in 500 µl buffer A supplemented with 1% (v/v) Triton X100 and broken at 4°C by vigorous shaking in a Fastprep-24 cell breaker twice for 20 s. The cell extracts were centrifuged at 20,000 × g for 30 min at 4°C to pellet the insoluble material. Labelled proteins were recovered by affinity chromatography in a Monomeric Neutravidin Resin (Thermo Scientific), with gravity flow, using phosphate-buffered saline (PBS, pH 8) + 1% Triton X-100 as equilibration and wash buffer. Proteins were eluted with an elution buffer (50 mM dithiothreitol (DTT), 2% SDS, 5% β-mercaptoethanol, 20% glycerol in 62.5 mM Tris–HCl, pH 6.8).

In the second extraction method, which is based on enzymatic shaving of surface proteins, biofilm cells were harvested by low-speed centrifugation (1,000 × g, 15 min, 4°C) to prevent cell lysis. The bacterial cell pellet was gently washed twice with 1 ml of ice-cold Tris Buffer Saline (TBS, 20 mM Tris-HCl pH 7.4, 150 mM NaCl). Pellet was resuspended in 1 ml of shaving buffer (20 mM Tris-HCl pH 7.4, 150 mM NaCl, 10 mM $CaCl_2$ $6H_2O$, 1 M L-arabinose) and bacteria were treated with 0.5 µg/ml sequencing grade trypsin (Promega, Charbonnières-les-bains, France) under gentle shaking at 37°C for 1 h. Bacterial cells were removed by centrifugation (1 000 × g, 15 min, 4°C) and the supernatant, containing trypsin-shaved peptides, was collected and filtered (0.22 µm). Digestion of peptides was completed overnight with 0.6 µg of trypsin at 37°C. Purification and concentration of peptides were carried out using Sep-Pak C18 Plus Short cartridges (Waters), pre-equilibrated in two steps with 65% acetonitrile (ACN)/0.1% trifluoroacetic acid (TFA) and 2% ACN/0.1% TFA. Peptides were loaded onto the cartridges, washed with 2% ACN/0.1% TFA and eluted with 65% ACN/0.1% TFA. Purified peptides were dried with a speed-vacuum and resuspended in 20 µL of 2% ACN/0.1% TFA.

The third and last method, fractionation of bacterial cells, biofilms were washed twice in Tris-EDTA (TE, 20 mM Tris, 5 mM EDTA, pH 7). Pellet was resuspended in 1 ml TE, and bacterial cells were broken using a cell disrupter (One shot cell disrupter, 1-8 ml, 2.7 KBar max, constant Systems Ltd, Daventry, UK) by applying 2.5 kBar pressure. Insoluble materials containing cell walls were removed by centrifugation (13,000 x g, 15 min, 4°C) and the supernatant was ultracentrifuged (200,000 x g, 1 h, 4°C). The supernatant corresponding to the intracellular fraction was saved and the pellet, containing membranes was washed twice in 1 ml Tris 40 mM, pH 8.5. Membranes were suspended in denaturing buffer (1% SDS, 0.1 M DTT, 20 mM Tris-HCl, pH 7.5) before heat-treatment (5 min, 95°C). Membrane and cell wall protein extracts were suspended in 100 mM ammonium bicarbonate pH 7.5. Proteins were reduced with 2.5 mM DTT at 56°C for 30 min, alkylated with 25 mM iodoacetamide during 20 min in the dark, and after digested overnight at 37ºC with 20 µg of trypsin (Promega) per sample. Extracted peptides were purified and concentrated with Sep-Pak C18 Plus Short cartridges as described above.

Comparison of protein extraction methods

The highest number of unique identified proteins by MS containing at least two unique peptides was obtained by the cell fractionation method (910, including both cell wall and membrane subfractions), followed biotinylation and shaving methods, 141 and 98 identified proteins, respectively (Figure 1A). The same assessment can be made in Figure 1B with all the results obtained from the three fractions of the cell fractionation method. There is a noticeable sharing of identified proteins between the three methods, and it reinforces that the highest number of unique identified proteins was obtained using cell fractionation, particularly the intracellular fraction. The identified proteins were annotated and separated accordingly to its predicted subcellular localization: intracellular proteins, membrane-associated proteins, cell wall-associated proteins and extracellular proteins. One of the aims of this study was to identify the highest number of surface-associated proteins possible, thus the use of three complementary extraction methods. However, despite the precautions taken to avoid cell lysis, a substantial number of cytoplasmic proteins was present in all extractions, particularly in the shaving and biotinylation methods (Figure 2). The highest percentage of intracellular proteins in proteosurfaceome subfractions was found in trypsin shaving (82%), followed by the wall, membrane fraction and biotinylation, corresponding to 75%, 53% and 41% of the total identified proteins, respectively. It is noteworthy that the biotinylation method had the lowest number and percentage of identified cytoproteins. Nonetheless, the membrane and wall

fraction allowed to access a higher amount of surface-associated and extracellular proteins. Regarding the cell fractionation, the intracellular fraction leads to the identification of a relevant number of cytoplasmic proteins (700 identified proteins). Concerning the cell membrane-associated proteins (CMAP), 157 were identified, the majority being once again retrieved by the fractionation method (Supplementary Figure 1A). As for the CWAP, cell wall-associated proteins, and extracellular proteins, 8 and 32 proteins were identified, respectively (Supplementary Figure 1B and 1C). In any case, the results highlight the complementarity of the methods used to extract surface-exposed proteins.

Protein extraction turnover

The three temperature settings chosen for this study were (i) 10°C, close to the refrigeration temperature used throughout the food industry chain and also in the consumer's home (Cabrita et al., 2013), ii) 25°C, an average ambient temperature of saprophytic life and (iii) 37°C the optimal growth temperature for Listeria and also the human host temperature (parasitic way of life) (Ivy et al., 2010). Globally, the number of proteins we identified was significantly higher than in the recently published paper of our group in which the three protein extraction methods were optimized to study *L. monocytogenes* proteomes (Esbelin et al., 2018). Cytoplasmic proteins had the highest percentage on the pool of identified proteins (79%), regardless of the methodology used for cell surface proteome extraction and the measures used to avoid cell lysis. This was a somewhat expected result since the majority of the cell proteome is composed by cytoplasmic proteins, and among them ribosomal proteins which account for more than 20% of the total cell proteins (Ryzhov and Fenselau, 2001). At the technical level, despite all the taken precautions, extraction methods can lead to cell lysis, resulting in a significant number of intracellular proteins being released (Quan et al., 2013). Furthermore, from the set of proteins characterized as cytoplasmic by *in silico* analysis, some may have a double localization and/or exhibit an additional function when localized at the bacterial cell surface. Moreover, it is feasible that the predicted subcellular localization of proteins may be incorrect or incomplete because they potentially harbour unknown motifs for cell-envelope attachment or their secretory pathway is not yet known (Esbelin et al., 2018).

Cabrita, P., Batista, S., Machado, H., Moes, S., Jeno, P., Manadas, B., Trigo, M.J., Monteiro, S., Ferreira, R.B., and Brito, L. (2013). Comparative analysis of the exoproteomes of *Listeria monocytogenes* strains grown at low temperatures. *Foodborne Pathog Dis* 10**,** 428-434.

Esbelin, J., Santos, T., Ribière, C., Desvaux, M., Viala, D., Chambon, C., and Hébraud, M. (2018). Comparison of three methods for cell surface proteome extraction of *Listeria monocytogenes* biofilms. *OMICS: A Journal of Integrative Biology*.

Ivy, R.A., Chan, Y.C., Bowen, B.M., Boor, K.J., and Wiedmann, M. (2010). Growth temperature-dependent contributions of response regulators, sigmaB, PrfA, and motility factors to *Listeria monocytogenes* invasion of Caco-2 cells. *Foodborne Pathog Dis* 7**,** 1337-1349.

Quan, S., Hiniker, A., Collet, J.F., and Bardwell, J.C. (2013). Isolation of bacteria envelope proteins. *Methods Mol Biol* 966**,** 359-366.

Ryzhov, V., and Fenselau, C. (2001). Characterization of the protein subset desorbed by MALDI from whole bacterial cells. *Anal Chem* 73**,** 746-750.
