# Supplementary material for: Listeria monocytogenes Biofilm Adaptation to Different Temperatures Seen Through Shotgun Proteomics
Source: Front Nutr. 2019 Jun 14;6:89. doi: 10.3389/fnut.2019.00089 (PMC6587611; doi:10.3389/fnut.2019.00089)
Supplement: Supplementary file 6 [file Data_Sheet_2.PDF]

Supplementary data captions

Supplementary Figure 1 – A: Venn diagrams representing the distribution of the 920 unique proteins identified by method of protein extraction; B: Same representation but with the added information about the different fractions of the fractionation.

Supplementary Figure 2 – Pie charts illustrating the number of identified proteins and the efficiency of each protein extraction method taking into account the predicted subcellular localization of these proteins.

Supplementary Figure 3 – A: Distribution of the Cytoplasmic membrane-associated proteins (CMAP) by extraction method in which they were identified; B: Cell-wall-associated proteins (CWAP) by extraction method in which they were identified; C: Extracellular proteins by extraction method in which they were identified.

Supplementary Figure 4 – A: Protein-protein map interaction of the 196 proteins that were more abundant at 10°C; B: Protein-protein map interaction of the 184 proteins that were more abundant at 37°C.

Supplementary Figure 5 – A: Protein-protein map interaction of the 212 proteins that were more abundant at 10°C; B: Protein-protein map interaction of the 124 proteins that were more abundant at 25°C.

Supplementary Figure 6 – A: Protein-protein map interaction of the 104 proteins that were more abundant at 25°C; B: Protein-protein map interaction of the 129 proteins that were more abundant at 37°C.

Supplementary Table 1 – Times when the biofilm was harvested taking into account the temperature and stage of growth.

Supplementary Table 2 – Complete account of the 380 statistical significantly different proteins obtained from the comparison between 10°C and 37°C.

Supplementary Table 3 – Complete account of the 336 statistical significantly different proteins obtained from the comparison between 10°C and 25°C.

Supplementary Table 4 – Complete account of the 233 statistical significantly different proteins obtained from the comparison between 25°C and 37°C.
