# Supplementary figures and images for: Listeria monocytogenes Biofilm Adaptation to Different Temperatures Seen Through Shotgun Proteomics
Source: Front Nutr. 2019 Jun 14;6:89. doi: 10.3389/fnut.2019.00089 (PMC6587611; doi:10.3389/fnut.2019.00089)

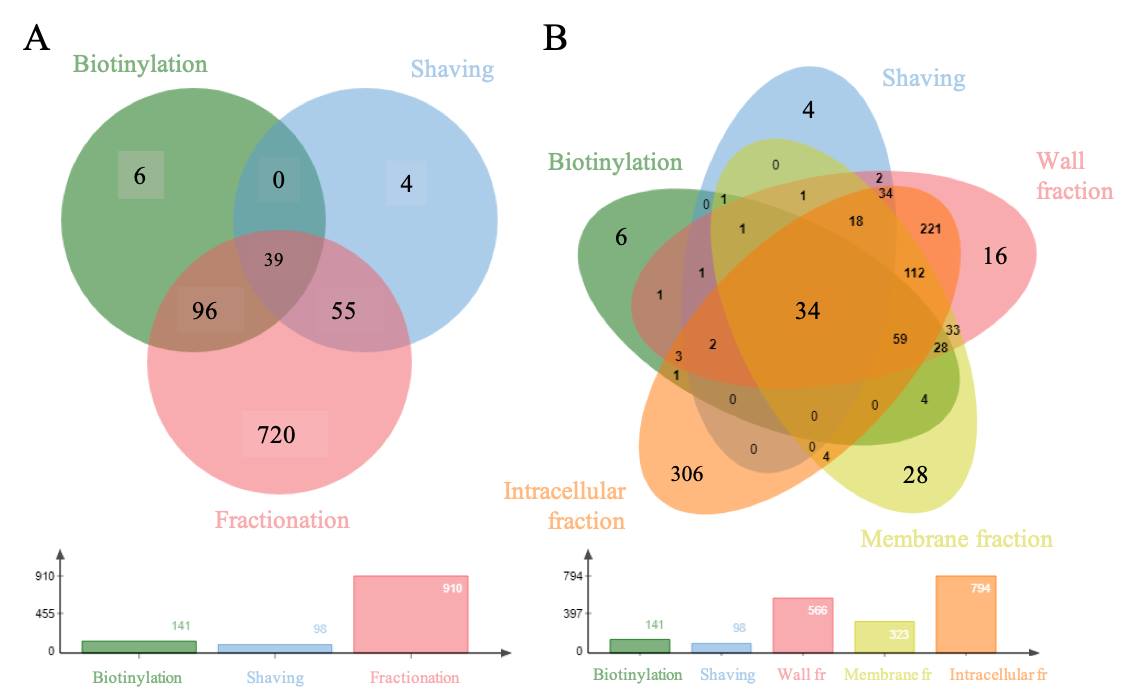

Supplement: Supplementary file 7 [file Image_1.JPEG]

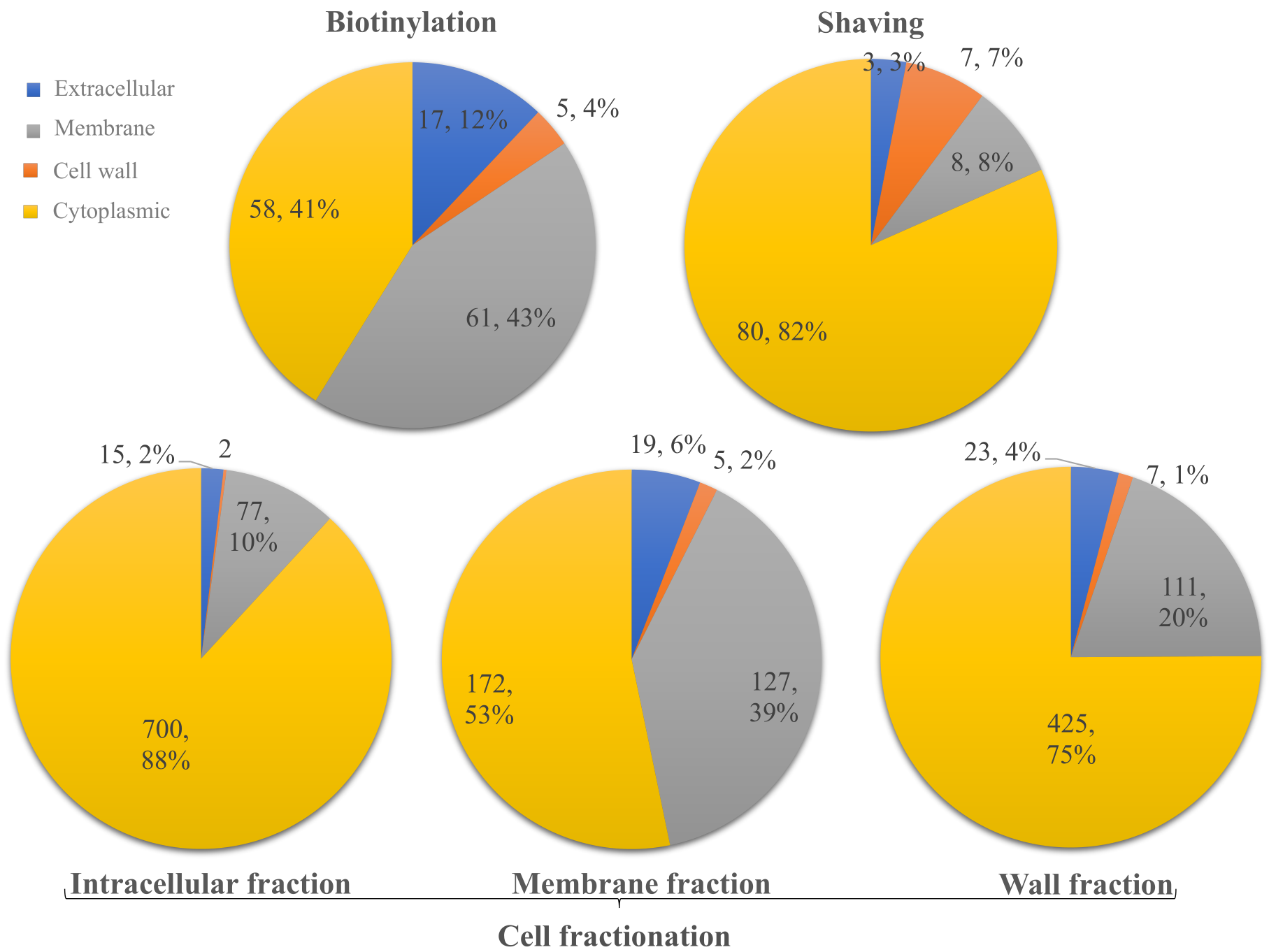

Supplement: Supplementary file 8 [file Image_2.TIFF]

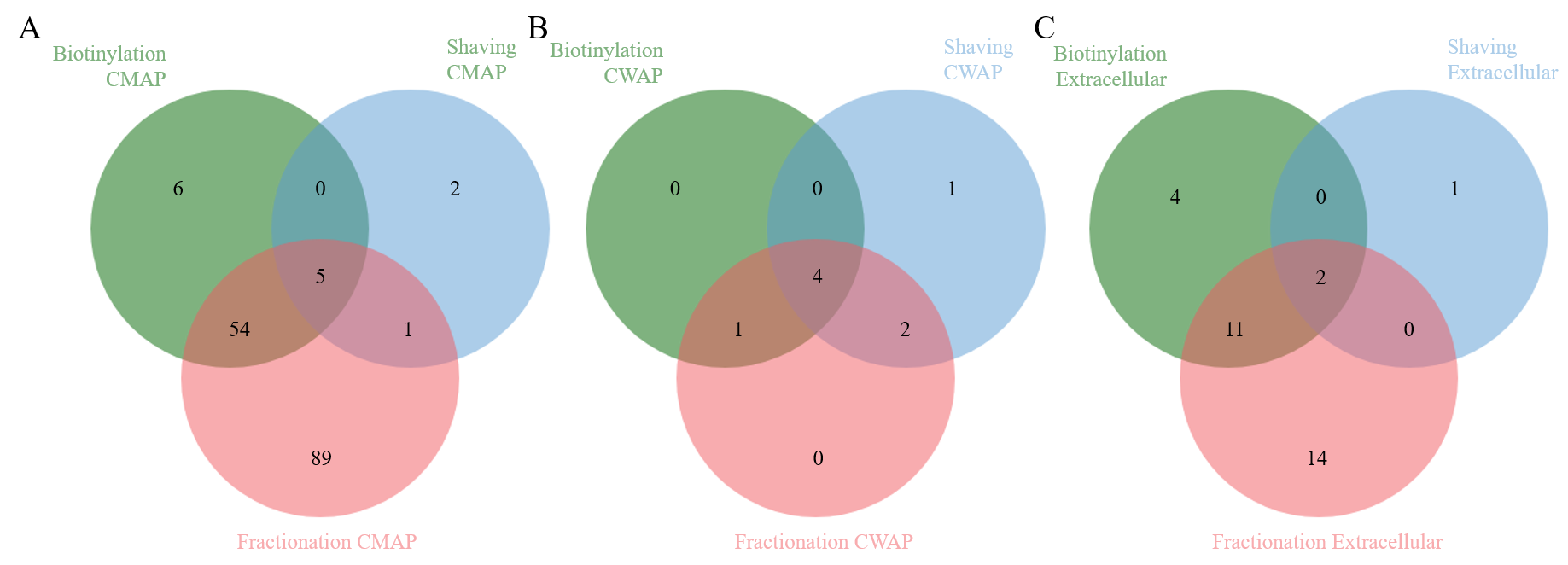

Supplement: Supplementary file 9 [file Image_3.TIF]

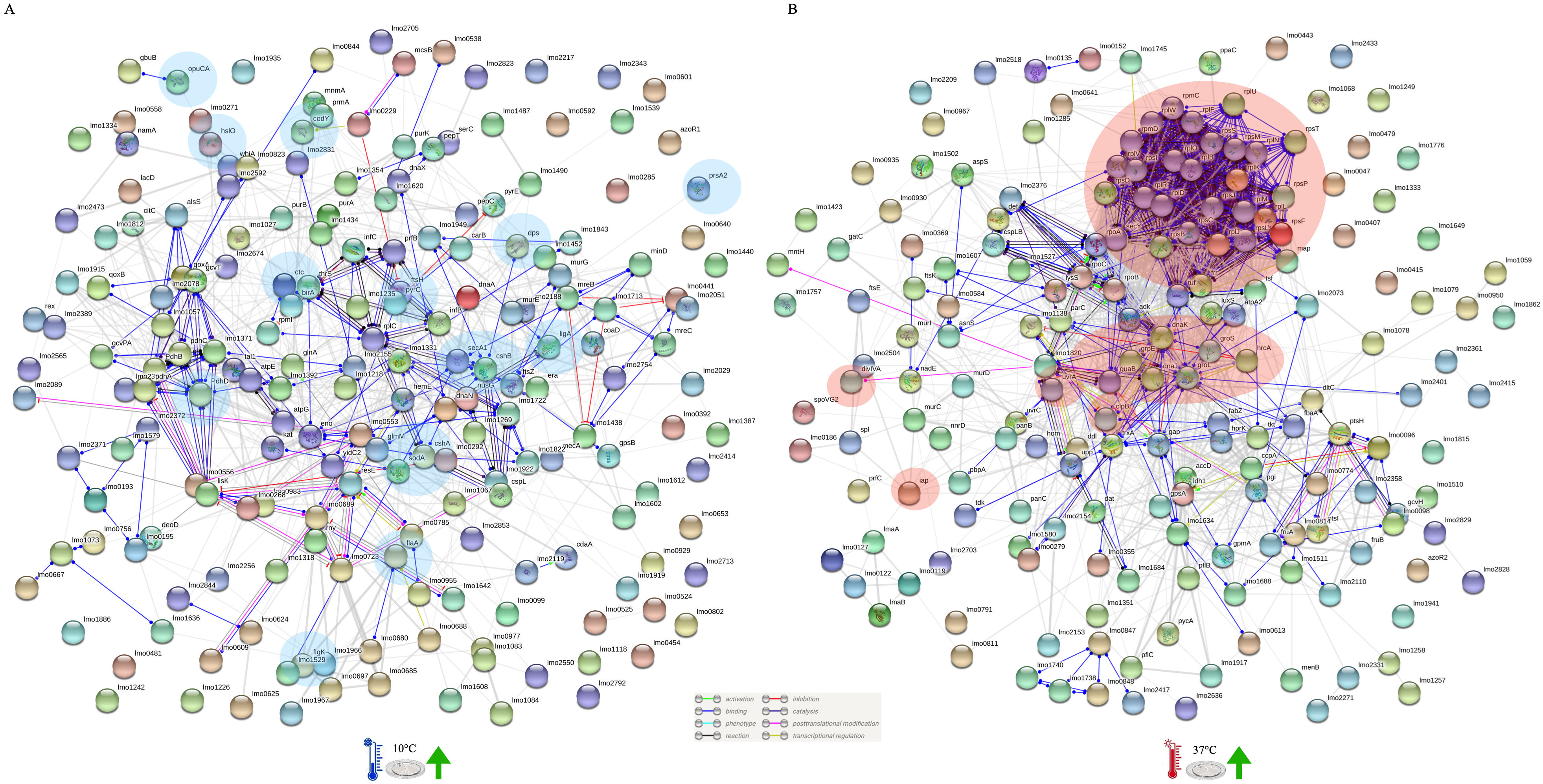

Supplement: Supplementary file 10 [file Image_4.TIFF]

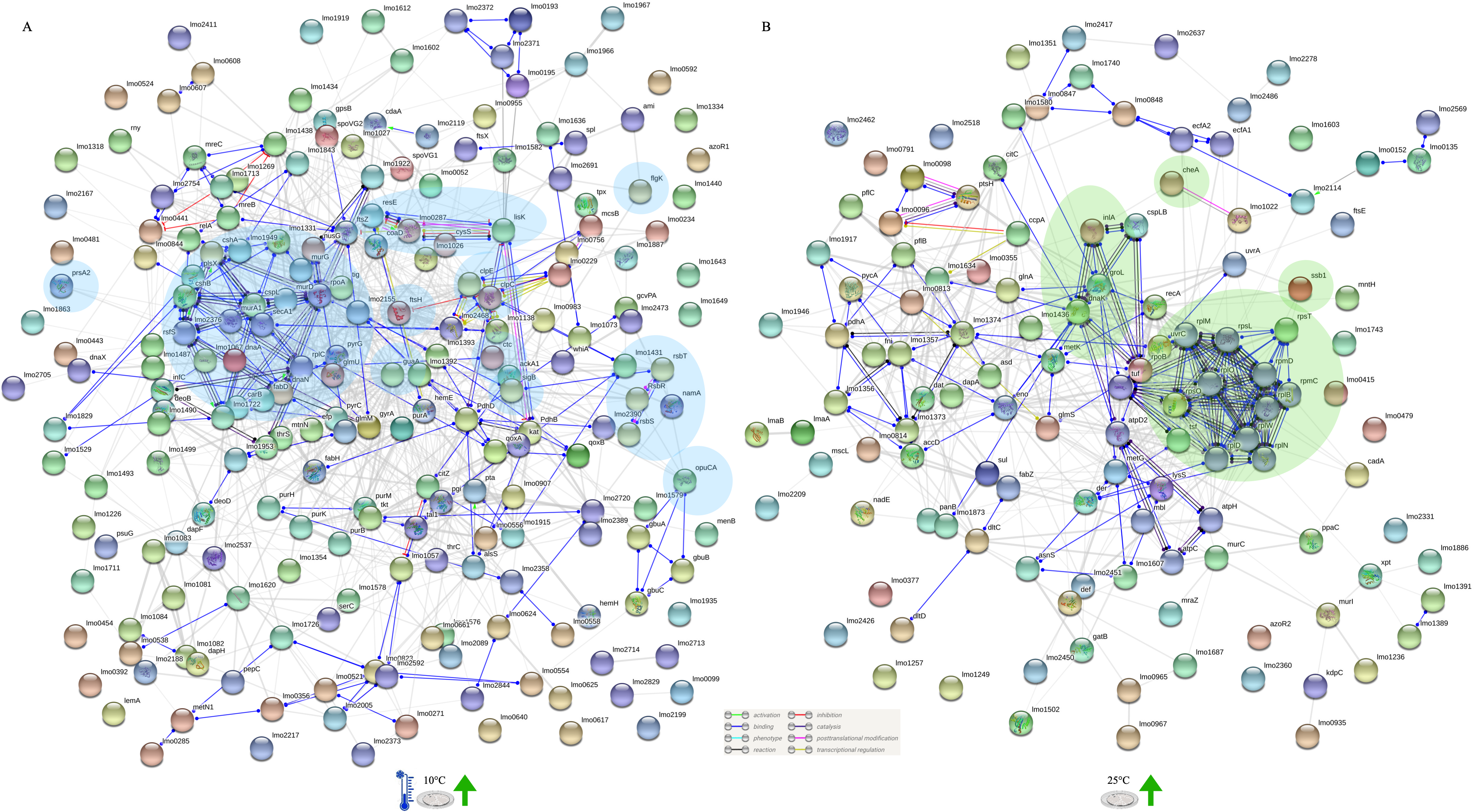

Supplement: Supplementary file 11 [file Image_5.TIFF]

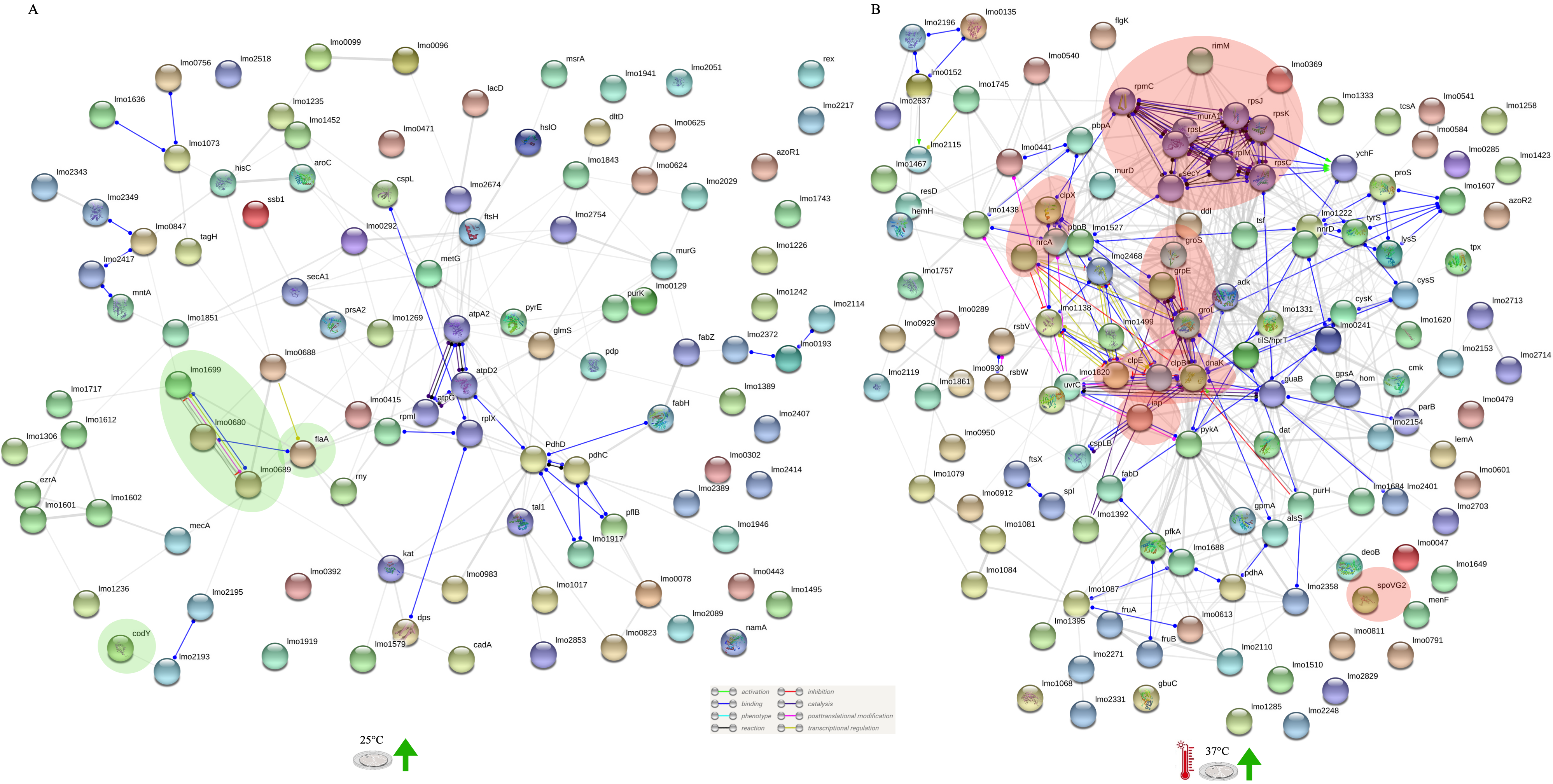

Supplement: Supplementary file 12 [file Image_6.TIFF]
